# Improving breastfeeding support through the implementation of the Baby-Friendly Hospital and Community Initiatives: a scoping review

**DOI:** 10.1186/s13006-023-00556-2

**Published:** 2023-04-15

**Authors:** Aisling Walsh, Pieternella Pieterse, Nita Mishra, Ellen Chirwa, Maria Chikalipo, Chimwemwe Msowoya, Ciara Keating, Anne Matthews

**Affiliations:** 1 RCSI, University of Medicine and Health Sciences, Dublin, Ireland; 2 Dublin City University, Dublin, Ireland; 3 University of Limerick, Limerick, Ireland; 4 Kamuzu University of Health Sciences, Blantyre, Malawi

**Keywords:** Breastfeeding, Baby-Friendly Hospital Initiative, Baby-Friendly Community Initiative, Scoping review

## Abstract

**Background:**

Improved breastfeeding practices have the potential to save the lives of over 823,000 children under 5 years old globally every year. The Baby-Friendly Hospital Initiative (BFHI) is a global campaign by the World Health Organization and the United Nations Children’s Fund, which promotes best practice to support breastfeeding in maternity services. The Baby-Friendly Community Initiative (BFCI) grew out of step 10, with a focus on community-based implementation. The aim of this scoping review is to map and examine the evidence relating to the implementation of BFHI and BFCI globally.

**Methods:**

This scoping review was conducted according to the Joanna Briggs Institute methodology for scoping reviews. Inclusion criteria followed the Population, Concepts, Contexts approach. All articles were screened by two reviewers, using Covidence software. Data were charted according to: country, study design, setting, study population, BFHI steps, study aim and objectives, description of intervention, summary of results, barriers and enablers to implementation, evidence gaps, and recommendations. Qualitative and quantitative descriptive analyses were undertaken.

**Results:**

A total of 278 articles were included in the review. Patterns identified were: i) national policy and health systems: effective and visible national leadership is needed, demonstrated with legislation, funding and policy; ii) hospital policy is crucial, especially in becoming breastfeeding friendly and neonatal care settings iii) implementation of specific steps; iv) the BFCI is implemented in only a few countries and government resources are needed to scale it; v) health worker breastfeeding knowledge and training needs strengthening to ensure long term changes in practice; vi) educational programmes for pregnant and postpartum women are essential for sustained exclusive breastfeeding. Evidence gaps include study design issues and need to improve the quality of breastfeeding data and to perform prevalence and longitudinal studies.

**Conclusion:**

At a national level, political support for BFHI implementation supports expansion of Baby-Friendly Hospitals. Ongoing quality assurance is essential, as is systematic (re)assessment of BFHI designated hospitals. Baby Friendly Hospitals should provide breastfeeding support that favours long-term healthcare relationships across the perinatal period. These results can help to support and further enable the effective implementation of BFHI and BFCI globally.

**Supplementary Information:**

The online version contains supplementary material available at 10.1186/s13006-023-00556-2.

## Background

Globally, improved breastfeeding practices have the potential to save the lives of over 823,000 children under 5 years old every year [1]. Exclusively breastfeeding infants for the first six months of their life is known to be the best start for a baby and a more widespread adoption of exclusive breastfeeding (EBF) would lead to the largest infant mortality reduction [1]. It can contribute towards meeting Sustainable Development Goals (SDG) 2 and 3—targets on nutrition and health—as well as being linked to many other SDGs. Since 1990, the World Health Organization (WHO) recommends that all newborn babies are exclusively breastfed for the first six months of their lives and continue to be breastfed for up to two years. Currently, 44% of infants under 6 months are being exclusively breastfed and just 35 countries are on target for exclusive breastfeeding [2]. Breastfeeding rates are both supported and hindered by the social determinants of health and multi-level support is needed, including at policy, health systems and services level, targeting communities and families [2–4].

The Baby-Friendly Hospital Initiative (BFHI), launched by WHO and United Nations Children’s Fund (UNICEF) in 1991, has been implemented globally in over 150 countries and is a pillar of the WHO/UNICEF Global Strategy for Infant and Young Child Feeding [4]. One of the nine operational targets of the *Global Strategy for Infant and Young Child Feeding* is to ensure that every maternity facility practices the BFHI’s ‘Ten Steps to Successful Breastfeeding’. Hospitals or maternity facilities can be designated “Baby-Friendly” if they pass an external examination that verifies that they comply with the Ten Steps to Successful Breastfeeding and with the ‘International Code of Marketing of Breastmilk Substitutes’ and subsequent relevant World Health Assembly resolutions (the Code). Table 1 details the Ten Steps, which were updated and revised in 2018, leading to a greater emphasis on scaling up to universal coverage, ensuring sustainability, and integrating the programme more fully with health-care systems [5]. Although the BFHI has been widely implemented, coverage at a global level remains low. In 2017 (the latest available data), just 10% of infants in the world were born in a facility currently designated as “Baby-friendly” [5].

**Table 1:** Ten Steps to Successful Breastfeeding [5]

**Critical management procedures:**	
**1a**	Comply fully with the *International Code of Marketing of Breast-milk Substitutes* and relevant World Health Assembly resolutions
**1b**	Have a written infant feeding policy that is routinely communicated to staff and parents
**1c**	Establish ongoing monitoring and data-management systems
**2**	Ensure that staff have sufficient knowledge, competence and skills to support breastfeeding
**Key clinical practices**	
**3**	Discuss the importance and management of breastfeeding with pregnant women and their families
**4**	Facilitate immediate and uninterrupted skin-to-skin contact and support mothers to initiate breastfeeding as soon as possible after birth
**5**	Support mothers to initiate and maintain breastfeeding and manage common difficulties
**6**	Do not provide breastfed newborns any food or fluids other than breast milk, unless medically indicated
**7**	Enable mothers and their infants to remain together and to practice rooming-in 24 h a day
**8**	Support mothers to recognise and respond to their infants’ cues for feeding
**9**	Counsel mothers on the use and risks of feeding bottles, teats and pacifiers
**10**	Coordinate discharge so that parents and their infants have timely access to ongoing support and care

The Baby-Friendly Community Initiative (BFCI) is an extension of the BHFI’s 10^th^ step of the Ten Steps to Successful Breastfeeding and of the BFHI overall [6]. Its focus is on community-based breastfeeding supports for women. Given the usual short postpartum stay in facilities, this 10^th^ step and associated separate initiatives are often critical to support breastfeeding mothers beyond the initial days of giving birth. While almost all countries in the world have implemented the BFHI at some point in time [4], it appears that the BFCI has been adopted in a smaller number of countries, namely low- and middle-income countries (LMICs), including Kenya, Cambodia, Gambia [6] and High Income Countries (HICs) such as Italy [7] and the UK [8].

There have been a number of attempts to review the literature on the BFHI [9–12]. Most of these reviews are dated and used inclusion criteria that resulted in these studies focusing on between 44 [9] and 58 [11] articles. Our review specifically sought to capture all evidence from a wider range of sources and settings and up to the current date.

This scoping review asks the question: what is known about the implementation of the BFHI and the BFCI globally? The aim is to map and examine the evidence relating to the implementation of BFHI and BFCI globally. Review objectives include:

1. To provide an overview of interventions and/or approaches to implement the BFHI/BFCI
2. To identify barriers and enablers to implementation of the BFHI/BFCI
3. To identify knowledge gaps in relation to research on the BFHI/BFCI

## Methods

Scoping reviews map the range of evidence on a particular topic, identify gaps in the knowledge base, clarify concepts, and document research that informs and addresses practice [13]. This scoping review has been conducted according to the Joanna Briggs Institute (JBI) methodology for scoping reviews [14]. We used the framework for scoping reviews developed by Arksey & O’Malley [15] as the foundation, updated and advanced by Levac et al*.* [16] and progressed further by new guidance from the JBI [14, 17]. According to this framework, there are six different stages, including: 1) identifying the research question; 2) identifying relevant articles; 3) study selection; 4) charting the data; 5) collating, summarising and reporting results; and 6) consulting with stakeholders. The scoping review has adhered to the Preferred Reporting Items for Systematic Reviews and Meta-Analyses Extension for Scoping Reviews (PRISMA-ScR) to ensure rigour in reporting. A protocol was published for this review [18].

### Stage 1: identifying the research question

A pilot search of the literature and scoping exercise was undertaken by our research team to examine empirical studies that have focused on the implementation of the BFHI in Africa [12]. During the literature search the following topics were examined: healthcare professionals’ knowledge and attitudes towards the BFHI [13, 15] compliance with the BFHI code [16] and the implementation of the BFCI [7, 8, 17]. At this time, we decided to focus on conducting a more systematic scoping review that incorporates both LMICs and HICs, in order to provide up to date evidence and to identify knowledge gaps.

### Stage 2: identifying relevant articles: search strategy

A three-step search strategy, as documented in the JBI manual was followed. Step one was a limited search for peer-reviewed, published papers on the PubMed and CINAHL databases. An academic research librarian was consulted and an analysis of the words contained in the titles, abstracts and index terms generated a list of keywords. Search terms were then piloted to assess the appropriateness of databases and keywords. The second step was conducted with the librarian which involved refining the search terms. The third step was to examine the references of key articles that were identified for full text review that met the inclusion criteria. Draft inclusion and exclusion criteria were tested on a sample of 15 articles to check the criteria’s suitability. The following databases were selected in consultation with the academic librarian: PubMed, Embase, Web of Science, Global Health and CINAHL. The timeframe for the search was from when the first article was published in a given database, which was 1993, to September 2022.

### Inclusion and exclusion criteria

Inclusion criteria were guided by the Population, Concepts, Contexts approach [16], as shown in Table 2.

**Table 2:** Population, Concepts, Contexts

**Criteria**	**Determinants**
Population	Women are pregnant, postnatal period and up to 2 years postpartum
Concepts	Baby-Friendly Hospital Initiative or the Baby-Friendly Community Initiative
Context	Hospital or community. No country or geographic location excluded

All research designs were included: qualitative, quantitative and mixed method studies. Quantitative studies included both experimental (e.g., randomised trials, non-randomised trials) and observational (e.g., cohort, cross-sectional) study designs. Qualitative studies included designs such as grounded theory, ethnography, phenomenology, action research and qualitative descriptive design. In addition, all types of reviews of empirical research were included. Grey literature was not included, due to the large numbers of results that were obtained. A full list of search terms is detailed in Additional file 1.

*Inclusion criteria*: *Articles that:*

- describe the implementation of the BFHI and/or BFCI
- evaluate the BFHI (any of the 10 steps) and/or the BFCI
- focus on experiences of accessing/delivering supports and services through the BFHI and/or BFCI
- focus on breastfeeding outcomes as a result of the BFHI and/or BFCI
- focus on any country or group of countries
- are in the peer reviewed literature
- empirical studies
- All types of literature reviews (e.g., systematic reviews, narrative reviews, scoping reviews)

*Exclusion criteria: Articles that:*

- focus on other breastfeeding initiatives, supports/interventions in the hospital and/or community other than the BFHI/BFCI
- the site is a baby friendly hospital but the study aim/objectives are not focused on the implementation of the BFHI/BFCI
- are published in a language other than English
- commentaries, opinion pieces, editorials, evaluations, theses and book chapters and conference proceedings

### Stage 3: study selection

**Fig. 1:**
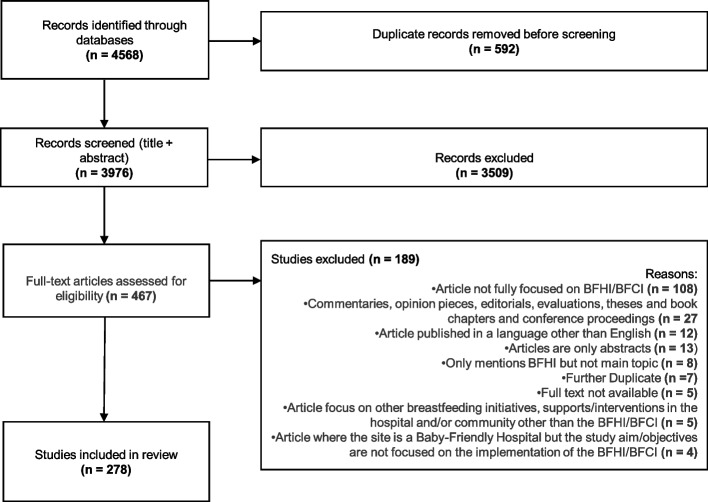
PRISMA Diagram

The screening process consisted of two phases: i) title and abstract screening; ii) full-text screening. In stage i) all titles and abstracts were screened by two reviewers in pairs (AM, PP, EC, MC, CM, AW). Screening was undertaken in Covidence and duplicates were removed. Where there was disagreement between reviewers as to whether an article should be included or excluded, a third reviewer arbitrated. At full text screening stage, the same process was undertaken. The original search was undertaken in 2020, an updated search was undertaken in 2022, and the overall results are shown in Fig. 1.

### Stage 4: charting the data

A data charting form was developed, piloted by all members of the team on five articles, amended and applied to all the included articles, according to the JBI framework [14, 17]. Data were charted in Covidence under the following headings: country, study design, setting, study population, BFHI steps, study aim and objectives, description of intervention, summary of results, barriers and enablers to implementation, evidence gaps, and recommendations. In keeping with scoping review methodology, an assessment of the quality of individual articles was not undertaken.

### Stage 5: collating, summarising and reporting result

It was originally planned to use the PAGER (Patterns, Advances, Gaps, Evidence for practice and Research recommendations) methodological framework [19] to analyse review findings, however, due to the high number of eligible studies, it was ultimately not practical to report the findings within this framework. However we have presented the findings of the review under patterns, evidence gaps and recommendations for practice to meet stakeholder needs.

### Stage 6: consulting with stakeholders

Findings from the review will be prepared for stakeholders who have expertise in relation to the BFHI and the BFCI. These will include researchers, practitioners and policy makers at the global level and at WHO regional levels.

## Results

### Characteristics of studies

The majority (*n* = 210) of studies focused on the BFHI overall/all steps, nine on the BFCI with 25 focusing on becoming BFHI/pre-BFHI (see Table 3). The vast majority of studies focused on hospital and community settings (*n* = 266), with five focused at the national level. In terms of study design, 46 were qualitative, 139 quantitative, six mixed methods, 28 reviews, and 39 intervention studies (see Table 4). Seventy-four of the studies were conducted in the United States of America, 18 in Brazil, 18 in Australia, 11 in Canada, 10 in the United Kingdom and nine in Italy (see Table 5). Most studies focused on mothers, babies or both (*n* = 144), with 60 studies focused on health professionals of various kinds (see Table 6). Sixteen studies were published between 1993 and 2000, 62 between 2001 and 2010, 169 between 2011 and 2020 and 31 across 2021 and 2022 (see Table 7). Additional file 2 provides an overview of all studies and their key characteristics.

**Table 3:** Number of studies as per 10 Steps

**Steps included**	**Number of studies**
BFHI overall/ All steps	210
BFCI	9
Becoming/ Pre BFHI	25
Neonatal BFHI	2
Step 1	2
Step 2	6
Step 3	5
Step 4	19
Step 5	9
Step 6	12
Step 7	11
Step 8	7
Step 9	12
Step 10	7

**Table 4:** Study designs

**Design**	***Which includes:***		**Number of studies**
Qualitative			46
Quantitative			139
	*‘Quantitative’*	11	
	*Census*	2	
	*Cohort*	10	
	*Cross sectional*	82	
	*Longitudinal*	8	
	*Prospective*	8	
	*Survey*	11	
	*Retrospective analysis*	7	
Mixed methods			6
Reviews			28
	*Review*	5	
	*Integrative review*	2	
	*Narrative review*	7	
	*Realist review*	1	
	*Systematic review*	13	
Intervention studies			39
	*Before and after*	12	
	*Intervention study*	1	
	*Non-randomised experimental*	11	
	*Quasi-experimental*	3	
	*Randomised*	12	
Case control/ case study			9
Economic evaluation			2
Evaluation			3
Other			6
TOTAL			278

**Table 5:** Country focus of studies

**Countries**	**No. of studies**
Belgium, Cyprus, France, Indonesia, Jordan, Korea, Mexico, Norway, Portugal, Puerto Rico, Republic of Belarus, Singapore, South Sudan, Sri Lanka	1
Austria, China, Egypt, Ghana, Greece, Iraq, Lebanon, Malawi, New Zealand, Russia, Sweden	2
Democratic Republic of Congo, Finland, Japan, Pakistan	3
Croatia, Iran, Malaysia, Nigeria, Saudi Arabia,	4
Hong Kong	5
India, Kenya, South Africa, Spain, Switzerland, Taiwan, Turkey	6
Included multiple countries	21
Italy	9
United Kingdom	10
Canada	11
Australia	18
Brazil	18
United States of America	74

**Table 6:** Population of study

**Population**	**No. of studies**
Postpartum women	76
Health workers	53
Postpartum women and newborns	47
Hospital or health facility level study	27
Newborn babies	21
Health workers, postpartum women (and babies)	18
Academic studies/other	9
Pregnant women, women attending antenatal care	6
Documentation review (usually of hospital/ health facility records)	5
Key BF stakeholders: lactation consultants, breastfeeding coordinators, hospital management	4
Postpartum women, health workers, and others involved in care (management, but also lay carers, etc.)	4
Health workers and hospital management	3
Medical student, recent medical graduates	2
Neonatal care staff/facilities	2
Pregnant and postpartum women	1

**Table 7:** Publication dates of studies

**Year range**	**Number of studies**
1993–2000	16
2001–2010	62
2011–2020	169
2021–2022	31

### Reviews

While we identified 28 reviews, there have been six comprehensive attempts to rigorously synthesise the evidence on BFHI. The most recent was a scoping review to identify challenges to the successful implementation of BFHI and explore strategies to overcome those barriers [9]. Our review differs in that it is wider in scope—has wider inclusion criteria—we have included 278 studies whereas Hirani et al. [9] included fourty-four. A systematic review by Perez-Escamilla and colleagues in 2016 [10] focused on the impact of the BFHI on child health outcomes up to 2012. This review concluded that adherence to the 10 Steps positively impacts early initiation of breastfeeding, exclusive breastfeeding and total duration of breastfeeding. Howe-Heyman & Lutenbacher [20] determined that the BFHI is an effective intervention to improve breastfeeding initiation, duration, and exclusivity. In addition to the various reviews, UNICEF has documented case studies of the experiences of 13 countries in implementing the BFHI, across high, middle and low/incomes countries [12]. There were also some more dated reviews [11, 21, 22]. Some reviews focus on specific steps, e.g. Step 2 [23, 24]. Some of the reviews are country specific, e.g. Korea [25], Australia, [26, 27], the US [28, 29] and the UK [8].

### Patterns

Initially, 13 patterns were identified in the extracted data. These were further analysed and most were found to align with a particular step of the ‘10 steps’ while others were more overarching, such as at national policy level. This alignment and the final six patterns to be presented are shown in Table 8.

**Table 8:** Patterns

	**Initial patterns identified**	**Revised patterns**	**Final pattern**
1	Policy (national) and health systems	Overall/ higher policy level	1 National policy/ system
2	Formula marketing	Step 1a—merged with hospital policy	
3	Socio-economic cultural factors	Merged with national policy	
4	Hospital policy	Step 1b plus most other steps	2 Hospital policy
5	NICU/pre-term	Step 7 plus others- merged hospital policy	
6	Becoming BFHI compliant	Pre-BFHI- merged with hospital policy	
7	Rates of BF	Overview of implementation outcomes (across steps)	3 Implementation of specific steps
8	Quantified implementation of 10 steps		
9	Baby Friendly Community Initiative	Step 10	4 Baby Friendly Community Initiative
10	Health worker knowledge and education/ training	Health worker factors: step 2	5 Health workers knowledge and education/training
11	Interprofessional collaboration		
12	Educational programmes for women	Step 3 plus many others- 8, 9	6 Educational programmes for women
13	Breastfeeding culture	Merged with women’s education	

#### 1. National policy and health system

In order to implement BFHI, many studies highlighted the need for effective and visible national leadership, demonstrated with legislation, funding and policy. At a national level, significance of legislation around the Code, executive and leadership support and culture, and providing adequate resources concerning uptake and implementation of the BFHI, was highlighted in an Australia-based study [30], which noted that little formal government support has been provided to further develop the BFHI and support the Code. Enablers such as endorsements of both local administrators and governmental policy makers, and effective leadership of the practice change process was noted in an integrative review across countries [11]. Strong recognition and support of the BFHI by the government was the most frequently reported facilitator at the socio-political level, while UNICEF-dedicated regional coordinators were singled out as political-level facilitators in Croatia [31], the USA and Brazil [32].

Specific **interventions** or approaches that were seen to be effective at a national level included: a national collaborative, run by the National Institute for Children’s Health Quality, that was designed to accelerate the number of Baby-Friendly designated hospitals in the United States [33]. Specific policy measures were also found to be important, such as, in Sweden which already boasts a strong overall pro-breastfeeding culture, the national health insurance system enabled mothers to spend as much time as they wished with their newborns in hospital [34]. In Brazil, it was identified as important to stimulate certain strategies through government policy, such as continuous internal evaluation, either through quality improvement projects or self-audits [35]. Also in Brazil, the provision of financial incentives to hospitals achieving BFH status was highlighted as important [36]. Promotion of social learning opportunities included ‘breastfeeding friendly premises’ to promote public breastfeeding; and for breastfeeding women to interact with antenatal women [37]. In New Zealand, improvements were achieved through the establishment of a national body with implementation and auditing oversight of BFHI facility, promoting Maori and consumer participation at all levels [38]. This study highlighted re-certification requirements for midwives of breastfeeding education by the Midwifery Council, and maternity facilities having paid BFHI coordinators were other enabling factors. Legislative efforts such as those in California (which requires all birthing hospitals to adopt the BFHI by 2025) were found to be effective at improving BFHI designation level [39].

**Barriers** seen at a national level in Australia, included lack of uniformity in perception of the benefits of BFHI at all levels of the health system, leading to varied uptake of the BFHI across the country [25]. Also in Australia, there were complexities and prolonged processes of accreditation and re-accreditation, which were linked to varied sizes of health facilities and geography [40].

##### Socio-economic factors

The range of issues identified under ‘socio-economic factors’ at the national level included, in terms of **barriers**, educational level of mothers in Russia [41], and in the USA: obesity [42], low wealth [43], ethnicity [39, 44, 45], and rural–urban residence [46]. Collaboration with government and other agencies and appropriate use of information technology were found to key **enablers** to improve outcomes within these studies. Those with higher education were more likely to commit to exclusive breastfeeding in Nigeria [47].

Language was identified as a barrier in Switzerland [48], which was mediated by the mother’s educational level and nationality. The term ‘socio-economic status’ is broadly used in studies, as impacting on the capacity of initiatives to increase breastfeeding rates, for example, in New Zealand [49]. Sometimes socio-economic factors were seen inter-sectionally, for example, rural-dwelling African American mothers were less likely to participate in BFHI initiatives [44]. Other studies showed higher rates of EBF in rural areas [46]. Ethnicity, specifically African-American or Hispanic ethnicity, was highlighted in the USA as being related to lower EBF rates [39, 45] though there can be challenges with data and records about ethnicity. Lisi and colleagues [50] studied the BF patterns on migrant mothers in Portugal and emphasised the sociocultural factors that influence practices, regardless of BFHI status of the hospital.

**Interventions** found to address these barriers include consistent and longer-term culturally tailored breastfeeding education, support, and equipment [29] and ensuring that language needs are met [48].

#### Pattern 2. Hospital policy (Step 1b, and affecting all other steps)

The issues raised in this pattern relate to the presence and visibility of a hospital policy and its implementation at hospital/ facility level, as well as wider hospital level activities to support BFHI implementation. Institutional support and strong infrastructure, including comprehensive written breastfeeding policies, are more likely to lead to/be related to better breastfeeding support services and better breastfeeding outcomes, in the USA [51] and Canada [52]. Only a small portion of BFHI hospitals that were examined in South Africa had a written breastfeeding policy (step 1b) [53]. Facilities (in the USA) that completed breastfeeding policy revisions improved their scores [54]. The shift from written breastfeeding and infant feeding protocols to formal signed breastfeeding policies in most maternity units was found to positively affect breastfeeding promotion in the USA [55]. Having administration staff commitment to BFHI and achieving accreditation and ongoing support was highlighted in Australia [56]. As well as the hard evidence from audits, training, statistics, a ‘hearts and minds’ approach, was seen to be valuable to emphasise feelings, meanings, attitudes and beliefs in the UK [57].

**Interventions** included having education materials distributed widely in institutions, and modified electronic records to prompt for BF interventions in the USA [58]. Free-of-charge staff education and on-site competency verification for intensively collaborating hospitals and low-cost education for all other hospitals, was found to have a positive effect in the USA [59].

**Barriers** included the lack of written policies themselves or poor communication about them. One study in Turkey showed that BF outcomes were impacted by a lack of hospital breastfeeding policy that was routinely communicated to health care staff [60]. A study of 49 Massachusetts health facilities where deliveries took place noted that most hospitals did not publicly display their breastfeeding policy for patients and staff to see [61]. Little awareness about the importance of having a written policy which reflects the 10 steps, was found in Missouri, USA [62]. Many hospitals in Malawi had not translated the policy into languages commonly spoken within the catchment area [6]. The barriers to implementing steps 1 and 2 in New Zealand included: hospitals at varying stages of BFHI policy development; hospital policy not necessarily based on government policy; hospital policies being communicated in differing ways and dependent on resources. Factors outside of hospital control impacted on capacity to improve breastfeeding rates in this same study. Practitioners beyond the direct jurisdiction and employment of the hospital posed additional challenges [49].

##### Preparation for BFHI accreditation

Several studies focused on the processes involved in preparing for BFHI accreditation and on their enablers. These were mostly in the USA, where information technology was seen as a key enabler, as well as having visual displays of the Ten Steps, a dedicated breastfeeding coordinator and cross-disciplinary champions [63]. Also in the USA, Feldman-Winter et al., [33] described a national collaborative and highlighted the importance of leadership, access to data, and the creation of front-line resources. In Croatia, additional enablers included adequate staffing and appropriate structures and policy as well as staff competency [64]. The need to publicise appraisal dates and to involve other private organisations and centres was highlighted in Taiwan [65]. Complexities of accreditation were noted as a barrier in Australia [39]. A study in the USA highlighted [66] the most challenging aspects as staff education, prenatal education for women, rooming-in arrangements, ensuring skin-to-skin contact and the ongoing use of pacifiers and bottles.

##### Neonatal care settings

Several studies in Canada, the USA and Australia focused on BFHI in the neonatal context at a hospital level. Important factors enabling neonatal implementation of BFHI, in one US study, were extra assistance for women separated from their infants (step 5), the creation of a breastfeeding-friendly environment and the removal of formula advertising from the hospital and the neonatal intensive care unit (NICU) (step 6), and the creation of a support system after the mother’s discharge (step 10) [67]. The overall BFHI guidance was criticised for not explicitly considering the NICU setting by Naylor et al. [68] in a USA context. Another study, from Australia, expanded on these NICU-related challenges by describing how women and their newborns were treated and considered separately so that their interdependence was difficult to maintain [69]. This separation was also highlighted by Benoit & Seminic [70] where the infant’s health, lack of breastfeeding support and knowledge were also factors that acted as barriers to BFHI implementation in Canada. An Expert Group from the Nordic countries and Canada suggested the addition of three guiding principles to support this vulnerable population of mothers and infants in the neo-natal care setting [71].

##### Formula marketing (Step 1a)

Studies that related specifically to infant formula marketing at hospital level were from Canada [52], the UK [72] Croatia [31], Saudi Arabia [73] and Brazil [74]. Older studies highlighted how free or heavily discounted formula and hospitals having exclusive contracts with formula companies undermined breastfeeding, while some hospitals practiced wider product endorsement by including infant formula in discharge packs. More recent studies highlight the role of ‘the Code’ in delaying weaning to counter aggressive marketing practices of the industry. Education included in the implementation of BFHI was linked with lower rates of formula feeding in Saudi Arabia [73]. Knutson & Butler [75] stated the need for community interventions to address misinformation about formula supplementation, especially in relation to addressing racial inequalities.

#### Pattern 3: implementation of specific steps

Overall, successful BFHI implementation was associated with higher rates of initiation and continuation of breastfeeding across studies [21, 76–78]. This applied across newborns at preterm and term gestations [79] in the USA. Overall, enablers within this pattern were found to include breastfeeding knowledge, inter-professional collaboration, specialised training and support groups. Barriers included poor infant health status, parent-infant separation, lack of parental involvement, low breastfeeding knowledge and low breastfeeding support.

Studies often measured which of the Ten Steps were fulfilled and concluded that the more Baby-Friendly hospital practices mothers met, the better the breastfeeding outcomes. This was found in Malawi [6] and Hong Kong [80, 81]. For example, a study in Hong Kong found that participants who experienced six baby-friendly hospital practices were significantly more likely to achieve their planned duration of breastfeeding than those who experienced one practice [80]. This is described by some as a ‘dose–response’ pattern, based on the number of steps implemented [82]. The cumulative effect of the BFHI practices, rather than each individual practice, was also found to be the most important in improving breast-feeding outcomes in the USA [83].

Some studies highlighted the importance of specific steps. Step 1 was found to be an important factor for exclusive breastfeeding duration in Turkey [84]. Staff shortages were found in Australia [56], Croatia [64], Pakistan [85] and Brazil, to affect steps 1, 5, 10 [74]. Step 3, antenatal education for women was found to be another very important factor for exclusive breastfeeding duration in Turkey [84].

Step 4, specifically skin-to-skin contact, was the focus of several studies, such as in Brazil [86] and the United States [87, 88]. Step 4 was hindered by the current maternity healthcare practice paradigm of mother-infant separation in the United States [86]. Caesarean section was found to be a persistent barrier to the early initiation of breastfeeding in South Africa [89], and specifically difficult in relation to steps 4 and 6 in Iran [90], Australia [69] and Italy [91]. A study in Croatia noted that training led to improved implementation of 4, Step 7, Step 8 in hospitals, though Step 9 was not implemented [64]. During COVID-19, rooming-in was seen to have increased in hospitals in Italy and most of the newborn care took place in the mother’s room [92]. This was also found in Spain [93]. However, it was reported in the Italian study that earlier discharge due to COVID-19 took away some professional support.

Hospital lactation policies, high rates of surgical deliveries and nurses having limited education in breastfeeding initiation best practices, were noted as barriers to best practices related to step 5 in breastfeeding initiation, in Colorado State, USA [94]. Step 6 was associated with a higher prevalence of exclusive breastfeeding in Brazil [95]. Step 7 was highlighted in Sweden [34] and Italy [91]. Negative staff perceptions were seen to decrease rooming in the UK in an older study [96]. Step 7 was highlighted in Korea [97], alongside antenatal education programmes, to increase exclusive breastfeeding (Step 3). Steps 7 and 9 were found to increase BF rates in Switzerland [98]. Steps 9 and 10 were associated with a higher prevalence of exclusive breastfeeding in Brazil [95].

A longer length of stay in hospital was seen as important to breastfeeding in Japan [99]. This duration of hospital stay was possible because of a lump-sum allowance for childbirth and nursing, in which the cost of delivery was covered by health insurance. This can be seen to positively affect steps 5–10. In Croatia, steps 1, 2, 3, 6, 10 were all referenced in a study examining pre- and post BFHI designation [64]; despite the apparent implementation success, only a minority of participants stated that their healthcare providers discussed infant feeding during pregnancy, and BFHI standards declined rapidly post-hospital designation, highlighting the need for regular monitoring and reassessment as well as ongoing, effective training for hospital staff. In a Canadian study, steps 2 and 10 were found to be most impactful [70]. Sometimes, unintended negative effects were found, for example related to step 10. The authors of a study in the Democratic Republic of Congo [100] compared participants in two groups, with steps 1–9 and steps 1–10 respectively, and found that having the 10th step actually lowered EBF rates. The authors speculate that perhaps the flyers shared with family members as part of step 10 were not appropriate in this setting and may have led to misinformation from family members to mothers, that were not countered by information shared by professionals on mothers’ visits to the clinics. On the other hand, Step 10 was seen as specifically important for specific under-served socio-cultural and economic groups in Australia [101].

Where studies measured the support for the 10 steps, there was considerable variation, for example, least support (28%) for step 1 and greatest support (93%) for step 3. Inconsistencies in implementation of the other steps were common in a study in the USA [62]. There was also variation in Iran [90] and Canada [102]. A USA study [103] reported adherence to the 10 Steps ranging from 10 to 85% (lowest for Step 9, highest for Step 10), with low adherence also to step 6 (do not provide breastfed newborns any food or fluids other than breast milk, unless medically indicated).

#### Pattern 4: the Baby Friendly Community Initiative (Step 10)

There was limited evidence about the Baby Friendly Community Initiative (BFCI), with just nine studies in total, focusing on Kenya, Italy and Turkey. In Italy, counselling or education being provided concurrently in various settings was seen to be most effective [104]. A national working group was also found to be important in Italy [104]. In Kenya, BFCI implementation had positive impacts on complementary feeding (weaning, dietary diversity) [105]. The national framework in Kenya included key enablers such as: capacity building, mentorship, integration, social mobilsation and supervision [6]. Integration was also highlighted as critical for Kenya [6, 106]. The community basis of Step 10 of the BFHI itself was seen to be critical for sustained improvements [10] and a lack of community services was seen to be a barrier to improvements [73].

#### Pattern 5: health worker knowledge and education/ training (Step 2)

Many different sorts of educational and training interventions were covered in the research. These were focused on improving both knowledge of breastfeeding, increasing support for the BFHI and improving attitudes towards breastfeeding [23, 24, 107, 108] across many countries. Training improved hospitals’ compliance with the Ten Steps in Italy [108] the UK [109] and Canada [110].

Knowledge of the BFHI varied across professional groups. The least understood steps in medical and nursing students were steps 1, 3, 8, and 10 [111] in India, with female students more aware than males about steps 2, 4, 5, 7 and 9. Knowledge was significantly lower among Residents than Specialists and nurses in Turkey [112]. In a study in Nigeria [113] only 20.8% of health workers were aware of the need to initiate breastfeeding within 30 min of delivery (step 4) and only 5.25% could demonstrate how to correctly position and attach a baby. In a nine-country study, four country case studies showed that whenever programmes were not attended by the majority of staff from all disciplines, there was resistance to change [114].

Time pressures, out of date practices and a lack of commitment to BFHI by experienced midwives was found to have a major impact on newly graduated midwives seeking to develop their breastfeeding support skills, in an Australia-based study [56]. In a USA study, 15% of staff did not understand the term “Baby-Friendly Hospital” when asked “Is your hospital a BFH?”, though 89% of consultants could answer this question [115].

***Enablers*** *:* Many studies emphasised the need to monitor ongoing learning. Some studies of interventions focused on single professions, for example nurses in Singapore [116], where training increased knowledge, including greater awareness of ‘the Code’. The influence of the nursing staff was greater than that of doctors [117] according to a study from Taiwan. A focus on basic skills was needed to improve confidence in the UK [109]. A study in the USA [118] highlighted the role of nurse-physician leadership dyads, who were committed to project goals, collaborative working and role modelling. The value of practical as well as theoretical training was emphasised in a study focusing in Texas, USA [119], where inconsistencies in practice and philosophies among nurses in how they approach breastfeeding support were reported. Better training resulted in fewer mixed messages in another USA study [72]. Continuous health education, in-service training, and teamwork amongst healthcare professionals were found to be key in South-Africa [120]. However, the additional cost of staff time for training was highlighted in a study in Indonesia [121]. The need for training and retraining to maintain compliance was emphasised in Hong Kong [122]. Gavine et al. [123] highlighted an urgent need for high quality research to inform the design and delivery of effective BFHI education and training. Improved self-reported knowledge was associated with those that have had children themselves and those who had formal breastfeeding education in Canada [124]. Lay breastfeeding educators/ counsellors were included in the focus in a review of the effectiveness of breastfeeding training in several countries [125].

**Barriers:** Overall, a lack of health professionals’ education was found to be a barrier to BFHI implementation across many studies. Inadequate training of health staff and a high volume of patients was a barrier in Pakistan [85]. A study in Kenya [110] highlighted specifically the lack of knowledge related to expressing milk, storing and treating expressed milk and emphasised related cultural barriers. Negative attitudes towards breastfeeding were found to be a barrier in Singapore [116] and Nigeria [126]. Health professionals in Australia expressed concerns about how the BFHI is misinterpreted by midwives and others and seen as something forced on women or reduced to being just a checklist [24]. Changing staff attitudes requires time, according to a study from Iraq [127], and similar findings were reported from Turkey [60] and the USA [61]. Whenever programmes were not attended by the majority of staff from all disciplines, there was resistance to change [114]. A study conducted in the USA concluded that staff may have been embarrassed to seek help if they felt lacking in a skill [58]. Poor communication by healthcare staff, including judgmental language and criticism of feeding attempts, affected women in the UK [8].

##### Interprofessional collaboration

Key **enablers** for BFHI implementation in the USA [66] were found to be linking multi-professional groups with interprofessional education and training, while in Spain, using a specific method of quality improvement cycles proved to be effective [128]. Both medical and nursing champions with shared values and vision were seen to be of great value in studies in the USA [118] and Canada [70]. It was reported that this type of interprofessional collaboration overcame isolated staff promoting breastfeeding [127]. A network for postnatal support was suggested, including lactation consultants and community groups, involving hospitals and outpatient clinics in Saudi Arabia [73]. The specific role of obstetricians supportive of breastfeeding was highlighted in Turkey [60], Taiwan [65] and in the USA [129]. Giving students who conducted work experience in South African public hospitals ‘roles’ within a breastfeeding support framework, such as health advocate, scholar, communicator, manager or professional, had a great influence on their awareness, which subsequently acted as catalysts for transforming practice [130]. Lactation consultants were seen as critical, providing both in- person and telephone support [131]. The involvement of hospital-based nutritionists and dieticians was seen as critical in Nigeria [132].

Various **barriers** such as the medicalisation of childbirth and inter-professional struggles were highlighted as hindering inter-professional teamwork and collaboration and, therefore, the implementation of BFHI and its integration into practice in Austria [133].

#### Pattern 6: educational programmes for women (Steps 3, 8, 9)

Many educational **interventions** for pregnant and lactating women were highlighted. Some interventions were Step specific, such as breastfeeding education in the prenatal setting, step 3 [63] and a training course for women on Step 4, in Egypt [134]. Mothers received education on both pacifier avoidance for breastfeeding and the use of pacifiers as a protective factor against Sudden Infant Death Syndrome, in the USA [135]. Objective testing of knowledge of breastfeeding support education in practice skills using self-study videos was undertaken in a study in the UK & China [136]. Confidence about breastfeeding was increased for women living in western Saudi Arabia through education and the introduction of strategies such as peer counselling [137]. Women who were in their first or second trimester of pregnancy until six months postpartum were offered a minimum of 12 personalised home-based counselling sessions on infant feeding by trained community health volunteers, in Kenya [138]. A breastfeeding ‘self-efficacy workbook’ for women in their third trimester improved breastfeeding self-efficacy and exclusive breastfeeding four weeks postpartum in Japan [139]. Other examples of enabling educational interventions included:

- providing sufficient information for mothers and the public about the BFHI, the benefits of breastfeeding, disadvantages of not breastfeeding, and benefits of going to accredited facilities (Australia) [40].
- mothers who gave colostrum as the first food had more frequently taken lactation counselling support than mothers who gave prelacteal foods (Turkey) [140].
- a client-focused practice development approach was found to be effective in Australia [141].
- viewing short videos increased breastfeeding knowledge, particularly about hand expression, and increased confidence in both skills (UK and China) [136].
- greatest improvements in breastfeeding were seen when counselling or education were provided concurrently in various settings [104].

***Barriers*** *:* A study in Cyprus found that a large proportion of pregnant mothers received limited information and/or education on the benefits and ways to achieve exclusive breastfeeding [142]. Administrative pressures or lack of support may have impaired some aspects of BFHI implementation in individual hospitals that were included in a study in New Hampshire, USA [59]. Cultural beliefs were shown to negatively influence pregnant mothers’ decisions regarding adopting good practice in breastfeeding in Nigeria, e.g. perception of colostrum being stale milk that stayed in the breast for the nine months of pregnancy [132]. In Lebanon, the majority of pregnant women who were surveyed for a study were unfamiliar with the terms baby friendly hospital, skin-to-skin contact or kangaroo care [143].

Cultural beliefs of mothers, their family members and others were seen as important across studies, as a wider context for BFHI implementation. A study in Iran [144] found that while breastfeeding is culturally the norm, exclusive breastfeeding is not, with supplements given to most newborns in hospital. This was also found in the USA [129] especially for those from under-served populations, of lower socio-economic status.

### Evidence gaps

Many studies identified gaps in the existing evidence. Patterns in study design and data collection that arose were as follows:

- *Study design issues:* The importance of conducting studies with a control group and the need to carry out more experimental studies was highlighted multiple times [20, 136, 145–147]. More cost-effectiveness studies are also required, as highlighted in a recent study [148]. Ducharme-Smith et al. [103] highlighted that there is still a need for studies using larger samples to robustly test for differences in practices associated with BFHI and to examine implementation of all steps among different groups of women. The lack of long-term documentation, longitudinal studies or a historical data series of external evaluations was noted in several articles [149–151]. Staff surveys carried out over time need to take account of turnover of staff, therefore longitudinal studies are recommended [116]. Mäkelä et al. [152] also emphasised the need to study the sustainability of achieved changes in practice, based on a study in Finland, as did a study in the USA [153]. Shing et al. [122] highlighted the need to consider wider cultural changes about breastfeeding, when conducting longitudinal studies. Multi-centre studies to investigate the impact and factors affecting the implementation of the BFHI programme are also needed [73].

- *Data issues:* There was a stated need to improve the quality and validity of the collation of breastfeeding data (e.g., by means of a standardised external review) and to perform prevalence studies. Limited size of samples was noted several times [55, 86, 154, 155]. Numerous studies [54, 156, 157] suggest standardised and expanded data collection methods and tools to measure breastfeeding rates, and validated collection instruments to obtain more accurate, reliable, and replicable results. In addition, self-reporting/ selection biases were pointed out in several studies [59, 158–160]. Shaker et al. [161] noted potential institutional-level confounders, suggesting obtaining responses from multiple individuals at each institution, and validating the hospital staff responses with individual patient responses to similar questions.

### Recommendations for practice and policy

Within many studies, recommendations for practice and policy were highlighted, across different levels and settings. At a national level, political enforcement of, and support for, BFHI implementation can assist in expanding the designation process of Baby-Friendly Hospitals [26, 76, 162]. National standards were seen as critical [162]. The inclusion of the principles and tools of the BFHI into national standards for healthcare facility accreditation was also deemed important [162]. There was a need to evaluate and recognise incremental improvements in breastfeeding-related maternity care practices [54]. Extending BFHI implementation to the private sector was recommended [163]. Support for hospitals not currently on the path to Baby-Friendly status may include evidence of positive results achieved in already accredited facilities to promote the uptake of these practices, and work toward achieving Baby-Friendly designation [164]. National awareness campaigns were also recommended [143].

Advocacy for additional government resources is needed to support scale-up of BFCI [148], alongside an assessment of the scaling-up environment for BFCI in the context of BFHI, using the Becoming Baby Friendly Index at subnational levels. BFCI indicators should be integrated into the routine community reporting tools and the District Health Information Systems for sustainability [6]. Future efforts should link implementation of BFCI and BFHI, per the updated WHO Guidelines [5], to ensure the continuum of care for breastfeeding counselling and support, from facility to community level [6]. A need for community involvement in the provision of appropriate support was recognised, with community support groups and community-based counsellors. Encouraging the formation of mother-to-mother support groups was recommended [165]. Future strategies focus on having all relevant community providers achieve Baby-Friendly Community Initiative accreditation [38]. Wider use of the World Breastfeeding Trends Index and the Becoming Breastfeeding Friendly Index [166] would add to the scale-up of BFHI. Culturally sensitive breastfeeding education is needed [29, 167] and considerations for the socio-economic status of a community, as well as the need for language translation services [49]. A study in Jordan highlighted the need to study how infant gender affects women’s breastfeeding choices and practices [168]. In LMICs, diversification of funding sources and partnerships for BFHI should be explored to ensure continued funding, with increasing government commitments [6].

Ongoing quality assurance is crucial, as is systematic (re)assessment of BFHI designated hospitals [64, 76, 169–174] in particular, integrating reassessment into quality improvement and strengthening capacity for quality assurance. New assessment tools may be used to precisely assess the difficulties experienced with BFH compliance requirements and to provide a database for future BFH reassessments [170]. Assessment should include socio-demographic data to understand the impact including on mental health related outcomes [175], and the impact that the BFHI has on disadvantaged or minority populations [176]. In addition, future quality improvement initiatives involving the BFHI should use a more robust reporting system that is automatic, less burdensome and integrated into electronic health records with the potential to enhance continuous quality improvement in maternity care practices [176]. Late preterm infants and their mothers may benefit from adaptation of Baby-Friendly Hospital practices, particularly in Level 2 and Level 3 NICU settings as the clinical situation permits [80]. Applying more proactive measures would have an even bigger impact on breast-feeding prevalence in the NICU [177].

At the hospital level, there were many recommendations highlighted. Hospital administrators should establish and monitor breastfeeding policies [80, 178, 179]. Updating and strengthening undergraduate, pre-service, and in-service breastfeeding support capacity and BFHI education and training for healthcare practitioners will ensure changes in practices over the long term [6, 65, 76, 121, 150, 170, 180, 181] including training of hospital managers [149]. Targeted educational interventions are needed [182].

Hospitals with Baby-Friendly status should consider models of breastfeeding support that favour long-term healthcare relationships across the perinatal period. There is a need for a continuous healthcare model [167], including post-discharge support services [101]. The importance of antenatal contact was emphasised [183, 184]. Consideration should be given to a combination of systems with attention given to the provision of support to breastfeeding mothers in the early weeks after birth [185]. To promote breastfeeding, interventions should be delivered in a combination of settings by involving health systems, home and family and the community environment concurrently [105]. As in other areas of public health, authors of a USA study emphasised that multiple strategies and actions across socio-ecological levels (e.g., government, institutions, intrapersonal, individual) are deemed to be necessary to change the political, administrative, and societal norms for maternity care practices and policies that support successful breastfeeding, both in the hospital and community settings [72].

## Conclusion

In this scoping review, we sought to identify and map what is known about the implementation of the BFHI and the BFCI globally. We have included evidence from a wider range of sources than before, across all settings. A limitation of the review is a lack of critical appraisal of included studies, which may have resulted in studies of low quality being included. Studies that were published in a language other than English were excluded. Evidence from over 48 countries globally, and gathered from many different stakeholders—from women, health professionals and policy makers—is presented and organised here, to highlight six key patterns associated with implementation of the initiatives. These patterns mapped, to some extent, on to the Ten Steps for Successful Breastfeeding themselves, and range from national health system level to community level interventions between women and health professionals and others supporting breastfeeding. The BFHI has been revitalised in many countries. It seems that the potential of the BFCI has not been realised in settings beyond the initial countries in which it was implemented. Evidence gaps highlighted the need for having longer term follow-up outcome data, and having experimental designs where appropriate.

These results can help to support and further enable the effective implementation of BFHI and BFCI globally. Researchers can build on this evidence base to plan and carry out higher quality studies to advance understanding and improve future implementation of the BFHI and BFCI.

## Supplementary Information

**Additional file 1.** Search terms.

**Additional file 2.** Overview of studies.

**Additional file 3.** Preferred Reporting Items for Systematic reviews and Meta-Analyses extension for Scoping Reviews (PRISMA-ScR) Checklist.

## Data Availability

All data generated during this study are included in this published article and supplementary information.

## Abbreviations

BFCI: Baby-Friendly Community Initiative
BFHI: Baby-Friendly Hospital Initiative
EBF: Exclusive Breastfeeding
HIC: Higher Income Countries
JBI: Joanna Briggs Institute
LMIC: Lower and Middle Income Countries
NICU: Neonatal Intensive Care Unit
PRISMA-ScR: Preferred Reporting Items for Systematic Reviews and Meta-Analyses Extension for Scoping Reviews
SDGs: Sustainable Development Goals
UNICEF: United Nations Children’s Fund
WHO: World Health Organization
