# Supplementary material for: Improving breastfeeding support through the implementation of the Baby-Friendly Hospital and Community Initiatives: a scoping review
Source: Int Breastfeed J. 2023 Apr 15;18:22. doi: 10.1186/s13006-023-00556-2 (PMC10105160; doi:10.1186/s13006-023-00556-2)
Supplement: Supplementary file 1 — Additional file 1. Search terms. [file 13006_2023_556_MOESM1_ESM.docx]

**Additional file 1: Search terms**

|  | **Pubmed Medline** |
| --- | --- |
| 1 | "Baby Friendly Hospital Initiative" OR BFHI OR "Baby Friendly Initiative" OR BFI OR "Baby Friendly Community Initiative" OR BFCI OR "Baby Friendly" OR "Ten Steps to Successful Breastfeeding" OR "10 Steps to Successful Breastfeeding" |
| 2 | Limit #1 to English language and 1991-2020 |
|  | **EMBASE on Embase.com** |
| 1 | "Baby Friendly Hospital Initiative" OR BFHI:ti,ab,kw OR "Baby Friendly Initiative" OR BFI:ti,ab,kw OR "Baby Friendly Community Initiative" OR BFCI:ti,ab,kw OR "Baby Friendly" OR "Ten Steps to Successful Breastfeeding" OR "10 Steps to Successful Breastfeeding" |
| 2 | Limit #1 to exclude 650 Medline records  #1 AND [embase]/lim NOT ([embase]/lim AND [medline]/lim) |
| 3 | Limit #2 to English language, 1991-2020 |

|  | **CINAHL on Ebscohost** |  |
| --- | --- | --- |
| 1 | "Baby Friendly Hospital Initiative" OR BFHI OR "Baby Friendly Initiative" OR BFI OR "Baby Friendly Community Initiative" OR BFCI OR "Baby Friendly" OR "Ten Steps to Successful Breastfeeding" OR "10 Steps to Successful Breastfeeding" | 1207 |
| 2 | Limit #1 to English, 1991-2020 | 1042 |
| 3 | Limit #2 to exclude Medline records | 675 |
|  | **Global Health on Ovid** |  |
| 1 | ("Baby Friendly Hospital Initiative" or BFHI or "Baby Friendly Initiative" or BFI or "Baby Friendly Community Initiative" or BFCI or "Baby Friendly" or "Ten Steps to Successful Breastfeeding" or "10 Steps to Successful Breastfeeding").mp. [mp=abstract, title, original title, broad terms, heading words, identifiers, cabicodes] | 536 |
| 2 | Limit #1 to English | 488 |
|  | **Cochrane Library** |  |
| 1 | "Baby Friendly Hospital Initiative" OR BFHI OR "Baby Friendly Initiative" OR BFI OR "Baby Friendly Community Initiative" OR BFCI OR "Baby Friendly" | 403  clinical trials |
|  | **Web of Science Core Collection** |  |
| 1 | TS=("Baby Friendly Hospital Initiative" OR “Baby Friendly Initiative" OR  "Baby Friendly Community Initiative") | 515 |
| 2 | Limit #1 to English | 498 |
